# Research progress on Chaihu Shugan San in treating perimenopausal syndrome: A review

**DOI:** 10.1097/MD.0000000000041044

**Published:** 2024-12-27

**Authors:** Xiaomeng Zhang, Lele Luo, Chenchen Wang, Wenjing Lv, Yuanfei Duan, Lingyuan Kong

**Affiliations:** a Shandong University of Traditional Chinese Medicine, Jinan, Shandong Province, China.

**Keywords:** Chaihu Shugan San, perimenopausal syndrome, research progress, review

## Abstract

Perimenopausal syndrome (PMS) refers to a series of physical and psychological symptoms caused by fluctuating or decreasing sexual hormone levels during the pre- and postmenopausal periods. With the rapid development of society, more and more women suffer from menstrual disorders and insomnia caused by PMS. Chaihu Shugan San (CSS) is a famous traditional Chinese medicine prescription known for soothing the liver, relieving depression, and regulating qi and blood. Numerous clinical experiments and pharmacological studies have confirmed that CSS has a significant effect on PMS treatment. However, the composition of CSS is complex, its pharmacological effects are diverse, and its therapeutic mechanism for PMS has not been clearly explained. Therefore, this article reviews the classical literature, mechanism, pharmacological effects and clinical research of CSS in the treatment of PMS, so as to provide a reference for clinical application and further research.

## 1. Introduction

Perimenopausal syndrome (PMS) refers to a series of physical and psychological symptoms in women who are pre- and postmenopausal (45–55 years old) due to fluctuations or declines in estrogen levels caused by the decline of ovarian function,^[1]^ such as hot flashes and night sweats, irritability, palpitations, insomnia or depression, forgetfulness, edema, loose stools, skin paresthesias, dizziness, backache, etc.^[2]^ According to epidemiological studies,^[3]^ the global incidence of PMS can reach 47.8%, with the highest incidence as high as 98%, which seriously affects women’s work and quality of life.

As a classic prescription of traditional Chinese medicine (TCM), Chaihu Shugan San (CSS) has a variety of active ingredients and has good efficacy in antidepressant, anti-inflammatory, and antitumor aspects. In addition, CSS can also regulate intestinal microbiota microorganisms and improve indigestion.^[4–6]^

In the treatment of PMS, CSS can improve PMS through a variety of mechanisms, which has the characteristics of multi-level, multi-pathway, and multi-target. For example, it upregulates the level of monoamine neurotransmitters, regulates the function of the hypothalamic-pituitary-adrenal axis(HPA axis), and upregulates estrogen receptor (ER) expression.^[7]^ However, at present, the pathogenesis of PMS has not been fully understood, and the specific molecular mechanism of CSS compound has not been systematically elucidated. Therefore, this paper combines the theories of traditional Chinese and Western medicine to systematically elucidate the etiology and pathogenesis of PMS, and makes a more comprehensive and systematic analysis of the pharmacological active ingredients that are effective in CSS, combined with clinical research, aiming to improve the pathogenesis of PMS and provide references for clinical and experimental research.

## 2. PMS pathogenesis and CSS ancient treatment records

### 2.1. The comprehension of PMS in TCM

Ancient medical books do not specifically record climacteric syndrome, but according to its clinical presentations, it can be categorized as “Menopausal disorders,” “Hysteria,” “Lily disease,” “Depression syndrome” in traditional Chinese medical science.^[8]^ These classification methods have a long history in TCM literature and are traced back to many ancient medical books. Su-wen: Naivety of the ancients^[9]^ says: “At the age of forty-nine, when the Conception Vessel and Thoroughfare Vessel experience a decrease in function and menstruation ceases, the woman becomes physically weak and incapable of becoming pregnant.” And Synopsis of Golden Chamber Women and Treatment^[10]^ profoundly discusses the dirty mania. Its symptoms are similar to the symptoms in PMS, which manifests as depression, fire burning Yin fluid, and so on. Fu Qingzhu’s Obstetrics and Gynecology^[11]^ is a female monograph from the Qing Dynasty. However, the book does not explicitly propose the modern medical term “PMS.” It elaborates on the variety of symptoms that women may encounter during menopause, such as menstrual disorders, hot flashes, excessive sweating, insomnia, and mood swings.

The etiologies and pathogenesis of PMS mainly lie in the gradual decline of kidney-qi, gradual deterioration of Tiangui, and deficiency of Conception Vessel and Thoroughfare Vessel. The Fu Qing Zhu Female Division^[12]^ further emphasizes the close relationship between the kidney and the liver: the kidney stores the essence and the liver stores the blood. When the Tiangui is depleted, the kidney essence is also weak, resulting in a lack of source of transformation of the blood essence, which causes liver blood deficiency and the inability to moisten and nourish other organs. These factors lead to the deficiency of blood and the imbalance of qi in the female body, which leads to the disorder of visceral function, especially the disparity Yin-Yang within the kidney. Deficiency of kidney-qi and progressive exhaustion of Tiangui are the basis of this disease, which is accompanied by excessive fire in the heart and liver. Yang deficiency of the spleen and kidney can be seen after a long period of time. PMS are the clinical manifestations of various syndromes, including liver and kidney Yin deficiency, kidney insufficiency, liver depression, heart and kidney disjunction type, and Xiaoyang moving sout.

In treatment, TCM^[13]^ focuses on the equilibrium of Yin-Yang within and outside the body. The treatment may include nourishing the liver and kidney to replenish Yin fluid, nourishing the kidney, soothing the liver to regulate qi-blood, strengthening the kidney and soothing the heart in order to achieve mental tranquility and clarity, or tonifying Yin-Yang to balance Yin-Yang in the body, so as to relieve the symptoms of PMS. Synopsis of Golden Chamber Women and Treatment^[14]^ asserts: Women exhibiting irritability, susceptibility to sadness, weeping, resembling possession by a spirit, frequently yawn, are treated with Gan Mai Da Zao Tang. The renowned formula from Shanghan Lun,^[15]^ Chaihu Jialonggu Muli Decoction, harmonizes the pivot, unblocks Yang, dispels heat, calms the mind, and equilibrates Yin-Yang. It can alleviate feelings of concurrent cold and heat, as well as both deficiency and excess, during the perimenopausal phase. Compilation of Medical Masters^[16]^ states that the Shu Gan Yi Shen Decoction is indicated for individuals experiencing stomach pain and constipation resulting from qi-blood deficiency. It is very efficacious in addressing renal deficit and hepatic stagnation associated with PMS. TCM has documented our knowledge of and approach to treating PMS, offering useful insights and a theoretical foundation.

### 2.2. The pathogenesis of PMS in modern medicine

In Western medicine, PMS is used to describe a set of symptoms that result from the failure of the autonomic nervous system. This dysfunction is caused by a decrease in ovarian function, hormonal imbalance, or a reduction in women before and after menopause. Clinical manifestations include menstrual disorders, hot flashes, sweating, irritability, palpitation, and insomnia.^[17]^ The hormonal changes associated with PMS primarily involve a decrease in ovarian function and disruption of the hypothalamic-pituitary-ovarian axis (HPO axis). The levels of estradiol (E2), luteinizing hormone (LH), follicle-stimulating hormone (FSH), and E2 fluctuate, resulting in an endocrine disorder. Bioinformatics results showed that 1 of the targets of PMS was mammalian sirolimus target protein (MTOR), silencing information regulator 1 (SIRT1), and P70 ribosomal S6 protein kinase (P70S6K), which are critical genes in the MTOR signaling pathway, located upstream and downstream of the pathway, respectively.^[18]^ These findings demonstrate a positive correlation between the expression level of SIRT1 protein and ovarian function, whereas a negative correlation is observed between the expression levels of p70S6K and MTOR protein and ovarian function. Studies^[19–21]^ have found that the expression of MTOR and p70S6K is reduced by the inhibition of the phosphatidylinositol 3-kinase (PI3K)/protein kinase B (AKT)/MTOR signaling pathway and that the tuberous sclerosis 1/2 (TSC1/TSC2) complex regulates MTOR signaling. They can affect the growth and proliferation of cells, improve ovarian function, increase the number of follicles, regulate the level of sex hormones, and alleviate a series of clinical symptoms produced by PMS.

Hormonal dysregulation in patients with PMS may contribute to decreased immune function, making women more vulnerable to infections and other diseases.^[22,23]^ It is found^[24–27]^ that interleukin-1β (Il-1β) and TNF α(TNF-α) can be inhibited by regulating the PI3K/AKT and mitogen-activated protein kinase (MAPK) signaling pathways. This can lead to improvements in the immunological function of patients. When neurotransmitters including β-endorphins (β-EP), dopamine (DA), norepinephrine (NE), and 5-hydroxytryptamine (5-HT) fluctuate in patients with PMS, symptoms such as sweating, sadness, sleeplessness, and hot flashes may occur. Studies have found that^[28]^ can enhance the expression of brain-derived neurotrophic factor (BDNF) by modulating neurotrophin signaling pathways, thereby increasing the expression of mitogen-activated protein kinase 1 (MAPK 1) in the hippocampus, and ameliorating discomfort symptoms such as insomnia and depression.

During free radical metabolism in patients with PMS, when malondialdehyde (MDA) levels rise, superoxide dismutase (SOD) and glutathione peroxidase (GSH-PX) activities decrease, which can upset the body’s free radical balance, leading to free radical metabolic disorders and cognitive impairment.^[29,30]^ It can restore ovarian function by regulating the PI3K/AKT/FOX O3A signaling pathway to inhibit oxidative stress and enhance the activity of SOD and GSH-PX in serum, while reducing the level of MDA content, the metabolism of free radicals, and the activity of SOD. This type of regulation can enhance free radical metabolism and SOD activity in the body, which is important for the treatment of PMS.^[31]^ In addition, the genes B-cell lymphoma-2 (Bcl-2), Bcl-2 related X protein (Bax), and caspase 3 (Caspase-3) have an impact on the death of ovarian granulosa cells. The decrease in the proportion of Bcl-2/Bax increases the apoptosis of ovarian granulosa cells, and it can suppress the production of Caspase-3 in ovarian cells by increasing the ratio of Bcl-2/Bax, regulating the extracellular signal-regulated kinase (ERK) signaling pathway, and down-regulating the transduction of caspase-dependent apoptosis pathway, 9 mRNA and expression of apoptosis protease activating factor 1 (Apaf-1) improved ovarian function and inhibited apoptosis of ovarian cells.^[32–34]^

In conclusion, imbalances in the endocrine system impair immunity, hasten apoptosis, cause oscillations in neurotransmitter levels, and interfere with free radical metabolism. Together, these factors affect the physical and mental health of perimenopausal women.

### 2.3. Exploration of the theoretical basis of TCM for treating PMS with CSS

As a fundamental formula within the enduring canon of TCM for addressing liver stagnation and regulating qi-blood flow, CSS plays a crucial role in modulating the body’s dynamics, reducing hepatic tension, and achieving a balance of qi-blood throughout the organism. It also effectively enhances blood circulation and alleviates discomfort.

While TCM literature does not clearly document the efficacy of CSS in treating PMS, it can successfully mitigate many pain symptoms associated with this condition. The “Qianzhai Medical Lectures”^[35]^ document states: CSS consists of Sini San augmented with Chuanxiong and Xiangfu to invigorate blood and regulate qi, with a specific focus on soothing the liver. In “The Golden Mirror of Medicine,” great emphasis is placed on its efficacy in pacifying the liver and relieving depression, making it suitable for cases of pronounced liver-qi stagnation. CSS^[36]^ significantly influences liver relaxation and alleviates depression while regulating qi-blood flow, which can address the syndrome of liver-qi stagnation that leads to functional dyspepsia. Consequently, CSS can mitigate the symptoms of PMS resulting from liver failure that leads to spleen and stomach issues. Whereas the “Complete Works of Jingyue”^[37]^ states: CSS alleviates pain in the flanks and ribs, accompanied by alternating chills and fever. And the “General Principles of Medicine” assert: CSS addresses liver damage resulting from anger, discomfort in the left hypochondrium, and blood stagnation above. CSS^[38]^ addresses ailments characterized by liver-qi stagnation, such as flank discomfort, and possesses properties that soothe the liver, alleviate depression, enhance blood circulation, and mitigate pain. Consequently, it may be utilized to alleviate symptoms such as depression, sleeplessness, and irritability resulting from liver-qi stagnation in PMS.

Upon rigorous textual analysis of TCM literature, it becomes evident that CSS possesses a profound theoretical grounding in TCM and boasts significant application potential. Studies on CSS offer novel perspectives and methodologies to contemporary medicine, enabling a deeper exploration of TCM’s academic and clinical utility.

## 3. Mechanisms of CSS in the treatment of PMS

CSS is a classical Chinese herbal formula with numerous known targets and diverse mechanisms of action. The mechanism of PMS treatment can be analyzed from multiple perspectives, including the effects on ERs, neurotransmitter regulation, and the regulatory effects of the HPA axis.

### 3.1. Estrogen secretion as well as receptor effects

Shen^[39]^ et al illustrate that CSS plays an ameliorative role in perimenopausal liver depression by increasing the expression of the ERs ERα and ERβ in the ovary and enhancing ER effects. This suggests that CSS is associated with the expression of ERs. In a study by Chen et al,^[40]^ the ER alpha/ER beta (ERα/Erβ) ratio is used as a key parameter, and it has been found that after the intervention of CSS, the ERα/ERβ ratio is restored to near normal levels. This suggests that CSS may correct endocrine disorders caused by fluctuating estrogen levels during perimenopause by adjusting the ratio of ERs, especially the relative expression of ERα to ERβ, and thus improves the symptoms of PMS associated with estrogen imbalance. However, the specific mechanism remains to be elucidated. A study by Wang et al^[41]^ illustrate that the treatment of patients with liver depressed polycystic ovary syndrome (PCOS) with the addition of CSS significantly improve sex hormone levels, including elevation of E2 levels, which suggests that CSS intervenes in the disease by regulating sex hormone levels. It is worth noting that ERs are not regulated independently, but work in conjunction with theneurotransmitters,5-HT, NE, and DA, which are the 3 main monoamine neurotransmitters that play key roles in regulating mood, sleep, and pain perception. Studies^[42,43]^ have shown that abnormal serum levels of 5-HT, NE, and DA in patients with perimenopausal syndromes are strongly associated with the development of clinical symptoms. Several of the above studies have similarly confirmed that estrogen therapy can significantly increase patients’ 5-HT, NE and DA levels, thereby alleviating clinical symptoms. However, the exact mechanism is unclear.

### 3.2. Neurotransmitter regulation

As a neurological disease, the therapeutic mechanism of perimenopausal syndrome cannot be separated from neurotransmitter regulation. Here we summary the regulatory mechanisms of monoamine neurotransmitters and pathways related to neurotransmitter regulation.

#### 3.2.1. Monoamine neurotransmitters

CSS has been shown in several articles modulate the levels of monoamine neurotransmitters such as 5-HT, DA and NE, all of which are associated with depressive disorders. 5-HT, which promotes pleasure and relaxing mood, is mainly produced in the of the intermediate suture, and tryptophan is its precursor. In the tryptophan-5-HT pathway, tryptophan hydroxylase 2 (TPH2) plays a rate-limiting role and TPH2 gene deletion leads to depression in mice.^[44,45]^ In the tryptophan-kynurenine (KYN) pathway, tryptophan(TDO) and indoleamine 2,3-dioxygenase(IDO) are important rate-limiting enzymes, with TDO metabolizing tryptophan mainly in the liver and IDO metabolizing tryptophan in other tissues. Excessive activation of the KYN-related pathway leads to tryptophan deficiency, which inhibits 5-HT synthesis and triggers depression.^[46]^ In addition, changes in 5-HT levels directly affect the function of 5-hydroxytryptamine receptor 1A (5-HT1A) and 5-hydroxytryptamine receptor 2 A(5-HT2A); 5-HT1A function is reduced and 5-HT2A function is enhanced in patients with depression. Paeoniflorin in CSS can reverse the expression of of aforementioned 5-HTIA and 5-HT2A receptors, and has antidepressant effects.^[47]^ Paeoniflorin can also inhibit TDO to exert antidepressant effects.^[48]^ CSS also exerts antidepressant effects by antagonizing 5-HT3 receptor signaling and maintaining the intracellular calcium ion balance. Monoamine oxidase (MAO) degrades 5-HT to 5-hydroxyindoleacetic acid (5-HIAA), and MAO activity is increased in the brains of depressed rats, additionally the components of CCS can inhibit MAO and alleviate depression.^[49]^ DA is a precursor of NE, and serum levels of DA and NE are low in patients with depression, whereas Chuanxiong volatile oil can increase the brain levels of NE and DA to exert antidepressant effects.^[50]^

#### 3.2.2. cAMP/Ca2 + signaling pathway

As a secondary messenger, cyclic adenosine monophosphate (cAMP) plays an important role in nerve conduction and emotion regulation.^[51]^ Chen et al^[52]^ detect mouse hippocampus using RT2 Profiler PCR Arrays microarray method after constructing a mouse model of liver depression and administering the drug, and find that after the intervention of CSS, there is a significant difference in the expression of genes related to the cAMP/Ca2 + pathway in hippocampal region of rats with liver depression evidence. Among them, the up-regulation of inducible nitric oxide synthase 2 (NOS2) may be related to hippocampal cell damage caused by depressed mood; the decreased expression of tyrosine hydroxylase (TH) may affect the synthesis of neurotransmitters NE and DA, whereas calmodulin 1 (CALM1) and s100 calcium-bingding protein G (S100G) are involved to the metabolism of calcium ions, and growth inhibitory stimulating hormone (SST) is related to neural conduction.^[53]^ This suggests that CSS affects the cAMP/Ca2 + signaling pathway in the hippocampal region, which improves depression and nerve conduction abnormalities in the liver depression syndrome of PMS.

#### 3.2.3. ERK1/2 related signaling pathway

Both upstream and downstream extracellular signal-regulated kinase1/2 (ERK1/2) mediate multiple depression-related pathways. In the context of perimenopausal syndromes, the identified ERK1/2-CREB-BDNF pathway ameliorates depressive disorders by mediating BDNF cellular activity, which promotes axonal growth and reduces central nervous aystem (CNS) damage.^[54]^ Liang et al^[55]^ reveal the mechanism: CSS can increase rat hippocampal ERK1/2, cyclic adenosine monophosphate -response element-binding protein(CREB), BDNF, and tyrosine kinase receptor B (TrkB) mRNA expression and protein levels, which in turn exert antidepressant effects. Chen et al^[56]^ find that the BDNF-TrkB-ERK1/2 pathway can also be activated by CSS, which improves the activity of BDNF cells.

### 3.3. HPA axis regulation

CSS improves the behavior of rats with perimenopausal syndrome and regulates the function of the HPA axis.^[57]^ Perimenopausal women are prone to HPA and gonadal axis dysfunction due to the fluctuation of hormone levels in the body, which in turn triggers depression symptoms such as low mood and anxiety.^[58]^ CSS is able to significantly reduce plasma cortisol (COR) and corticotropin-releasing hormone (CRH) levels, while significantly increasing adrenocorticotropic hormone (ACTH) levels, suggesting that CSS can effectively regulate the levels of key hormones in the HPA axis, thereby counteracting perimenopausal depression.^[59]^ Studies have demonstrated^[60]^ that female rats with liver depression syndrome have dysregulation of neuroendocrine function, which manifests as hyperfunction of the hypothalamic-pituitary-adrenocortical/thyroid/gonadal axis with impaired negative feedback regulation of the hypothalamus and/or pituitary gland.CSS is able to regulate the abnormality of the neuroendocrine axis in this rat model, and its effect is better in large doses than in small doses, suggesting that CSS has a potential advantage in regulating the neuroendocrine axis.

### 3.4. Immunomodulation and inflammatory response

Changes in sex hormones also affect immune system function, which may lead to increased inflammatory responses and oxidative stress.^[61]^ The modulatory effect of Chaihu Shuogan San on interleukin-2 (IL-2) reveals its influence on the immune system of rats with PMS hepatolysis syndrome. IL-2 is up-regulated in rats with hepatolysis syndrome and its expression is reduced after following treatment with CSS, which is similar to the effect of antidepressant drugs, indicating that CSS may reduce the inflammatory state associated with PMS hepatolysis syndrome and contribute to the improvement of the mood by suppressing excessive inflammatory response.

### 3.5. Other mechanisms

Other mechanisms have been demonstrated^[62,63]^ that CSS can effectively reduce the apoptosis rate of tongue epithelial cells in perimenopausal syndrome. The degree of liver depression in perimenopausal syndrome patients is significantly higher than that in healthy women. The severity of liver depression correlates with the maturation degree of tongue exfoliated cells, the apoptosis index, and the expression levels of apoptosis-regulating genes (factor associated suicide, Bax, and Bcl-2). This suggests that liver depression is 1 of the core etiologic factors of perimenopausal syndromes and is closely related to apoptosis of tongue shedding cells. The pathological changes of liver depression are positively correlated with the positivity of Fas in tongue exfoliated cells, positively correlated with the positivity of Bax, and negatively correlated with the positivity of Bcl-2. These changes in apoptosis-regulating genes reflect the effect of hepatic depression on apoptosis in tongue epithelial cells; Fas and Bax are key proteins that promote apoptosis, while Bcl-2 functions as an inhibitor of apoptosis. Therefore, regulating the expression of these genes helps reduce the apoptosis of tongue epithelial cells and improve the condition.

## 4. Pharmacological effects of CSS

The formula of CSS consists of 6 herbs: Chaihu, Paeonia lactiflora, Fructus aurantii immaturus, Chuanxiong, Xiangfu, and Licorice. Chaihu is found to be the most effective ingredient. The main active ingredients in Chaihu chinense are saikosaponins (saikosaponin A, saikosaponin D and saikosaponin flavonoids), which can regulate and alleviate perimenopausal syndrome. The polysaccharides contain in these ingredients also have hepato protective and immune-regulatory effects.^[64]^ In addition to Chaihu, the active ingredients contained in other drugs in CSS also have significant therapeutic effects on various organs such as the liver and kidneys through targeting mechanisms.

### 4.1. Chaihu

As the main ingredient of CSS, it plays a significant role in regulating the liver, relieving depression, and promoting qi flow. The main active ingredients in Chaihu are Chaihu saponins and Chaihu brass, which play a crucial role in the treatment of PMS. Lin et al^[65]^ find that SSa can activate the PI3K/AKT signaling pathway by improving insulin resistance, thereby reducing liver damage. Gu et al^[66]^ also conduct a research on SSa and find that it has a protective effect on the liver. Han et al^[67]^ find through experiments on the regulation of canine uric acid metabolism by SSd in depressed rats that SSd has a significant regulatory effect on the HPA axis and can treat depression. Chaihu flavonoids can weaken the phagocytic ability of macrophages, rapidly and efficiently eliminate hydroxyl radicals and superoxide anions, and enhance the body’s immunity.^[68]^ Chaihu also has multiple targets including tumor necrosis factor (TNF), mitogen-activated protein kinase 3 (MAPK3), and interleukin-6 (IL-6).These targets can inhibit HBV replication through the TNF signaling pathway, alleviate chronic inflammation in the liver, and reduce the damage caused by inflammation to the liver.^[69]^ In clinical practice, Chaihu is commonly used as a component in the treatment of coronary heart disease with anxiety and depression, exerting its effects of soothing the liver, relieving depression, regulating qi and calming the mind, and has good clinical value.

### 4.2. Chenpi

Chenpi plays an important role in the treatment of liver-qi stagnation by regulating qi, strengthening the spleen, drying dampness, and resolving phlegm. The main active ingredient is nobiletin (NOB). In the study of tryptophan metabolism,^[70]^ it has shown that tangerine peel is often used as the main TCM component in its research, and it frequently appears in formulas related to liver and spleen regulation. Including anti-inflammatory and antioxidant effects, especially in solid organ damage caused by various factors, Chenpi extract can exert organ protection by regulating different molecular targets. In the experiment of Tong Sen et al^[71]^ exploring the regulatory effect of NOB on platelet activating factor in rats with diabetes induced kidney damage, we found that NOB can reduce the damage of distal convoluted tubules, reduce the possibility of kidney function damage, and also play a role in regulating blood sugar. Another study has shown that the qi-regulating effect of tangerine peel can greatly reduce oxidative stress in high-fat mice,^[72]^ providing another development approach for its clinical role.

### 4.3. Chuanxiong

Chuanxiong is known as a “blood qi medicine” and its blood-activating and qi-regulating effects give it a potential mechanism for soothing the liver and regulating qi. Its main components are Chuanxiong volatile oil, ferulic acid, ligustrazine, and Chuanxiong polysaccharides. Chuanxiong volatile oil and ferulic acid can promote liver cell proliferation, reduce liver cell apoptosis, and thus alleviate liver fibrosis. Ligustrazine has the effect of regulating inflammation and preventing cell apoptosis. Chuanxiong polysaccharides can provide protection against brain and myocardial damage, as well as have antioxidant and immune enhancing effects,^[73]^ making them highly valuable for future clinical research. Sun et al^[74]^ find that the anti-inflammatory effects of Chuanxiong could improve liver damage. As a high-frequency liver meridian drug for treating migraine, it not only protects the nervous system, but also improves inflammation and oxidative metabolism.

### 4.4. Xiangfu

Xiangfu Chang is the main medicine used for treating liver-qi stagnation and chest and rib pain. Through the extraction of Xiangfu, it was found that it contains various important substances with pharmacological activity, such as α-cyperone (CYP), volatile oils, flavonoids, tea polyphenols, etc. Research has shown that CYP can exert neuroprotective effects and alleviate intestinal inflammation by inhibiting the production of inflammatory cytokines in mice.^[75]^ In a study^[76]^ of the antidepressant components and related targets of Xiangfu, Jia et al construct a “compound-core target-pathway” network and have found that 69 compounds in Xiangfu acted on 103 targets, 29 core targets, and it can fight depression through 8 related metabolic pathways, revealing the antidepressant effect mechanism of Xiangfu through the synergistic effect of multi-components, multi-targets and multi-pathways.

### 4.5. Citrus aurantium

Fructus Aurantii has an extremely high application value as a medication for treating qi disorders in both drug therapy and clinical treatment. In an experiment conducted by Zhao et al^[77]^ to study the material basis and mechanism of action of Fructus aurantii in reducing blood sugar, HPLC-Q-Exactive Eorbitrap MS/MS combined with network pharmacology technology is used. Various components of Fructus aurantii, such as rutin and hesperidin, have hypoglycemic effects and play an important role in protecting the liver. In addition, the flavonoid structures extracted from Fructus aurantii immaturus, such as glycosidic dihydroflavones, have better prostaglandin-endoperoxide synthase 2 (PTGS2) binding activity and anti-inflammatory and analgesic effects.

### 4.6. Chinese herbaceous peony

As a TCM that promotes the release of liver-qi and regulates emotional activities, peony plays an important role in modern Chinese medicine. Song Chunhong, Wang Jieqiong et al^[78]^ find the effects of peony extract on the hypothalamic signaling pathway in rats with liver-qi depression syndrome, which can inhibit the calmodulin/Ca2+/calmodulin-dependent protein kinase II (CaM/CaMK II) signaling pathway mediated by Voltage-dependent L-type calcium channel subunit alpha-1C(Cav1.2), provide protection to the nervous system, and treat mental disorders related to emotional disorders. At the same time, it can also treat kidney disease caused by spleen kidney Yang deficiency, improve edema, and delay renal function damage.^[79]^ Experimental evidence has shown that paeoniflorin can regulate the phosphatidylinositol 3-kinase(PI3K)/AKT/MTOR signaling pathway, significantly increasing the levels of 5-HT and NE in hippocampal and cortical tissues.^[80]^ Other studies have shown that^[81]^ paeoniflorin can reduce the phosphorylation levels of PI3K, AKT, and MTOR, inhibit the activation of the PI3K/AKT/MTOR signaling pathway, and in some cases, lower blood pressure.

### 4.7. Licorice

Licorice has the effects of clearing heat, detoxifying, relieving pain, and harmonizing various medicines. Wang Suru^[82]^ extracts and produces active drugs, mainly composed of glycyrrhetinic acid, glycine, and cysteine. These active ingredients not only relieve liver cirrhosis and liver damage but also play a major role in correcting the immune system and reducing the possibility of inflammation. Zhang Jianxin et al^[83]^ also find that glycyrrhizin extracted from licorice is a flavonoid compound that has neuroprotective effects, which can regulate neuronal apoptosis, alleviate nerve damage, and other effects. Yang et al^[84]^ confirm that licorice can regulate the gut microbiota and downregulate the expression of IL-1 β, IL-6, and TNF-α to alleviate liver damage (Table 1).

**Table 1:** Effective pharmacological ingredients and functions of single medicines in CSS.

Medicine	Active ingredients	Pharmachologic effects	Affects target organs and their effects
Chaihu	Chaihu saponins^[64]^ Chaihu brass^[64]^	Activate the PI3K/AKT signaling pathway.^[65]^ Targeted action TNF, MAKP3, IL-6^[69]^	Liver^[66]^ Playing a protective role, Alleviates chronic inflammation in the liver Reduces the damage of inflammation to the liver
Chenpi	nobiletin^[70]^	Anti-inflammatory, antioxidant,^[70]^ and reduces liver collagen deposition.^[71]^	Kidney^[71]^ Reducing renal function damage
Rhizome of chuanxiong	Chuanxiong volatile oil, Ferulic acid, ligustrazine	Anti-inflammatory effects,^[73]^ promoting hepatocyte proliferation, and reducing hepatocyte apoptosis^[74]^	Liver^[74]^ Improving liver damage
Xiangfu	α-Cyperone, Volatile oil, tea polyphenols, flavonoids^[75]^	Anti-inflammatory, antioxidant, analgesic.^[75]^	Intestine^[75]^ Relieving intestinal inflammation
Citrus aurantium	Rutin, hesperidin^[77]^	Hypoglycemic, anti-inflammatory, and analgesic effects^[77]^	Liver^[77]^ Playing a protective role
Chinese herbaceous peony	Paeoniflorin^[78]^	Inhibition of CaM/CaMK II signaling pathway^[78]^ Regulatingthe PI3K/AKT/mTOR signaling pathway^[80]^	Spleen and kidney^[79]^ Delaying renal injury function
Licorice	Glycyrrhizin, glycyrrhetinic acid, glycine, cysteine^[82]^	Downregulation of IL-1 β, IL-6, TNF - α,anti-inflammatory liver injury, and maintains the immune system.^[82]^	Liver^[84]^ Relieving cirrhosis and liver damage

Abbreviations: AKT = protein kinase B, APK3 = mitogen-activated protein kinase 3, CaM/CaMK II = calmodulin/Ca2+/calmodulin-dependent protein kinase II, CSS = Chaihu Shugan San, IL-6 = interleukin-6, IL-1β = interleukin-1β, MPI3K = the phosphatidylinositol 3-kinase, TNF = tumor necrosis factor, TNF-α = tumor necrosis factor α.

## 5. Clinical case reports and experimental studies on the treatment of PMS with CSS

PMS is mainly divided into 2 syndromes: liver-qi reverse flow and depression.^[85]^ TCM believes that the liver is in charge of dredging, regulating qi flow, and whether emotional activity is closely related to liver function. Currently, the incidence of PMS is on the rise, and the clinical symptoms are numerous and complex, and are related to the dysfunction of multiple systems of the human body. Modern medicine believes that PMS pathogenesis is closely related to gamma-aminobutyric (GABA), 5-HT, and other mechanism disorders.^[86]^ Therefore, the clinical treatment of PMS by CSS has mainly been studied in the nervous, digestive, and reproductive related system.^[87]^

### 5.1. Liver-qi depression

#### 5.1.1. Kidney deficiency liver depression

A middle-aged woman has experienced amenorrhea along with hot flashes and cold sweats for more than 3 months. Menstruation is dark red, no blood clots, sweating, irritability, emotional depression, and general joint pain discomfort. Light red tongue, thin white fur, thin pulse string. The main pathogenesis is kidney deficiency and liver-qi stagnation, resulting in loss of mental health and a series of dysfunctional symptoms. CSS soothes the liver and regulates menstruation, obviously improving the symptoms of poor sleep, lower quality of life, and abnormal mood during the perimenopause period.^[88]^ From the point of view of Western medicine, *Bupleurum chinense* has a good therapeutic effect by mainly realizing its effect of soothing the liver and relieving depression through multiple components, channels, and targets, thus improving neuroendocrine function.

Insomnia is the most common disease in the neurology department, and the CSS modulates the neuroendocrine system. Liver depression type insomnia mostly manifests as difficulty in falling asleep, restlessness, dreaminess, and easy waking.^[89]^ This type of insomnia is mostly caused by anger injuring the liver, losing its ability to reach, depression for a long time, and disturbance of the mind. Yang Defu et al^[90]^ find that after grouping treatment of patients with insomnia of the liver depression type, the sleep quality and depression state of a group of patients taking CSS are significantly better, and the symptoms manifested by liver depression are significantly improved. In summary, CSS can regulate liver-qi and treat insomnia.

### 5.2. Liver-qi invasion

#### 5.2.1. Stagnation of liver-qi

A 49-year-old female with liver-qi stagnation and fire-melting syndrome. Six months after hysterectomy, paroxysmal fever and sweating occur all over the body for more than 2 years and aggravates for half a year. The onset is unusually irritable, restless, sweating all over the body, cold, and skin rose. The onset occurs several times per day, with no fixed time. Western medicine is used to diagnose climacteric syndrome. In the past 6 months, the frequency of attacks gradually increased, and the degree was aggravated. Prescription: CSS and Chaihu Longgu oyster decoctions combined. All symptoms are resolved after medication.

PMS patients with the liver-qi inversion type are mainly characterized by irritability, accompanied by mild breast pain,^[91]^ hot flashes, and sweating. Moreover, qi depression is 1 of the most common physical characteristics in perimenopause women. The basic formula for CSS is modified. The whole prescription mainly uses pungent San to soothe the liver, supplemented by astringing and softening Yin. Dragon bones and oysters are used to suppress Yang and calm the mind, thus improving hormone levels.

From the perspective of Western medicine, CSS can regulate the hormone rhythm of the HPO axis, regulate GABA and other pathways to improve hormone levels, and regulate the balance of qi-blood, Yin, and Yang.^[60,92,93]^ After CSS treatment,^[94,95]^ the expression levels of 5-HT and other brain neurotransmitters are improved, the hormone level is improved, and the levels of TNF-α and IL-6 are effectively reduced, thus relieving anxiety and depression. Li Hong et al^[96]^ find that CSS can reduce the transformation score of qi depression constitution in perimenopause women, improve hormone levels and biased constitution of women, and regulate the balance of qi-blood, and Yin by treating perimenopause women with CSS oral granules.

#### 5.2.2. Stagnation of qi due to depression of the liver, sputum resistance knot

A 51-year-old female has been depressed and in a bad mood. After examination, the patient is diagnosed with perimenopause syndrome. Diagnosis: depressed spirit, sometimes upset, poor diet, heavy muscles all over the body, blocked breath in the throat, light tongue, white and greasy fur, and deep and stringy pulse. Syndrome differentiation includes liver depression and phlegm qi obstruction. Prescription: CSS and Banxia Houpu Decoction.

According to the disease manifestations, CSS promotes the dispersion of liver-qi and is supplemented by herbs that nourish and soften the liver, while Banxia Houpo Decoction promotes the circulation of qi, dries dampness, and resolves phlegm. The herbs in each formula interact with and complement each other.

The spleen is the source of phlegm production. The spleen governs transportation and transformation. When the body is invaded by dampness, or when there is excessive worry and anxiety, these factors can all harm the spleen and impair its function of transportation and transformation, leading to the accumulation of water-dampness internally and its coagulation into phlegm. Furthermore, the liver is the source of onset, the stomach is the site of disease transmission.^[97]^ CSS soothes the liver, relieves depression, regulates spleen and stomach qi, and normalizes its transportation and transformation. Hui et al^[98]^ treat patients with chronic atrophic gastritis with conventional oral Western medicine and modified CSS. The result shows that the main clinical symptoms disappeared in the observation group and the inflammatory reaction was improved by gastroscopy. Therefore, CSS can regulate the spleen and stomach qi, and thus, its transport function returns to normal (Table 2).

**Table 2:** Clinical case reports and experimental studies on the treatment of PMS with CSS.

Pattern of syndrome	Study	Subjects	Grouping	Effects	Results
Kidney deficiency liver depression	Yang et al.^[90]^	120 patients with liver depressed insomnia	Group1: Take orally Chaihu Shugan San 1 dose/d, 200 mL, 60 m before going to bed for 4 wk. Group2: Take orally esazolam tablets 1 mg/time, taken 60 m before bedtime and treated for 4 wk.	Regulating nervous and endocrine system	The total response rate is 91.7% and 66.7%, respectively (*P* < .05); it is better than the control group in Pittsburgh Sleep Quality Index (PSQI) score, Self-rating Depression Scale (SDS) score and individual symptom effectiveness. Chaihu Shugan San can regulate liver-qi and treat this type of insomnia.
Stagnation of liver-qi	Li et al.^[96]^	35 women with qi depression during perimenopause	Group1: Take orally Chaihu Shugan San once daily for 12 wk. Group2: No treatment is given and observed simultaneously for 12 wk.	Improving hormone levels	The transformation of the depression is decreased (*P* < .05), E2 and TH are increased, and FSH and LH levels are decreased (*P* > .05); there is no change in the control group. Chaihu Shugan San can reduce the transformation score of qi depression constitution in perimenopause women, improve hormone level and biased constitution of women.
Liver depression and phlegm qi obstruction	Zhang et al.^[98]^	100 patients with gastric chronic atrophic gastritis (CAG)	Group1: Chaihu Shugan San plus subtraction treatment, 1 dose per day, fried twice in the morning and evening on an empty stomach. Group2: Using conventional treatment with Western medicine.	Regulating Spleen and Stomach	The overall response rate is 94.0% and 70.0% in the control group. In the 2 groups, the difference is significant (*P* < .05). Chaihu Shugan San can regulate spleen and stomach qi.

Abbreviations: CAG = chronic atrophic gastritis, CSS = Chaihu Shugan San, FSH = follicle-stimulating hormone, LH = luteinizing hormone, PSQI = pittsburgh sleep quality index, SDS = self-rating depression scale, E2 = estradiol, TH = tyrosine hydroxylase.

## 6. Conclusion and Outlook

In recent years, many researchers have made significant advances and break-throughs in pharmacological research and clinical trials of CSS for treating PMS, while also investigating its medical benefits.

According to pharmacological research, the main mechanisms of CSS’S active ingredients in treating PMS include enhancing the ESR1 effect; regulating the levels of 5-HT, DA, and NE; regulating the function of the HPA axis; anti-inflammation; and monoamine neurotransmitter regulation. Thus, it can reduce nerve damage, resist PMS depression, and play an antidepressant role.

From a clinical perspective, CSS has a positive effect on PMS, which can improve the quality of life of perimenopausal women by improving the cardiovascular system, soothing the liver, and regulating qi, treating insomnia, regulating the qi of the spleen and stomach, and so on.

Although current research has been conducted on the use of CSS to treat PMS, there has not been much fundamental or thorough study. The mechanism mostly revolves around menopausal depression, insomnia, depression, hormone regulation, and other aspects, and the mechanism of the research results is not in-depth.

There are numerous clinical studies on the use of CSS in combination with other medications to treat PMS; however, there are few reports on the use of CSS alone for PMS treatment, and most of these studies deal with liver depression treatment. A-thorough, in-depth analysis of CSS is not possible owing to the relatively narrow research direction, which leaves clinical research lacking a strong experimental foundation.

Future studies should build on the fundamental research on CSS, target its primary mechanism of action, and broaden their investigation to include other PMS related functions such as immune regulation, oxidative stress management, and inflammation regulation. Additionally, their research should take into account the various PMS manifestations and treatment strategies based on available evidence; at the same time, we should make use of new technology to improve the level of research, multi-target research and treatment of diseases, and improve the persuasiveness of conclusions. To ensure better PMS treatment and prevention in the future, we can also investigate and treat PMS in accordance with various experimental conclusions, develop new targets and mechanisms of action, and enhance the clinical safety, efficacy, and rationality of CSS for the treatment of PMS.

## Acknowledgment

We gratefully acknowledge the constructive comments of the manuscript provided by Dr Feng and thank the editor and anonymous reviewers for their contributions to improving the integrity of this auricle.

## Author contributions

**Conceptualization:** Xiaomeng Zhang, Lingyuan Kong.

**Visualization:** Xiaomeng Zhang, Lingyuan Kong.

**Writing – review & editing:** Xiaomeng Zhang, Lele Luo.

**Writing – original draft:** Chenchen Wang, Wenjing Lv, Yuanfei Duan.
